# Development and temporal validation of a postoperative nomogram based on clinical, surgical, histopathological, and semi-quantitative EEG features for predicting seizure-free survival in children after focal epilepsy surgery

**DOI:** 10.3389/fped.2026.1869297

**Published:** 2026-08-28

**Authors:** Xiaoyan Dong, Ting Sun

**Affiliations:** Department of Pediatric Neurosurgery, Xinhua Hospital, Shanghai Jiao Tong University School of Medicine, Shanghai, China

**Keywords:** epilepsy surgery, interictal spike index, nomogram, pediatric drug-resistant epilepsy, seizure-free survival

## Abstract

**Objective:**

Accurate estimation of seizure recurrence risk after pediatric epilepsy surgery may support individualized postoperative surveillance and management. This study aimed to develop and temporally validate a prognostic nomogram for estimating 1-year and 2-year seizure-free survival after focal or lobar resection in children with drug-resistant epilepsy.

**Methods:**

This single-center retrospective cohort study included 256 children treated between 2015 and 2024. Patients treated during 2015–2021 constituted the training cohort (*n* = 181), whereas those treated during 2022–2024 constituted the temporal validation cohort (*n* = 75). Eighteen prespecified clinical, neuroimaging, scalp EEG, surgical, and histopathological candidate predictors were evaluated using least absolute shrinkage and selection operator penalized Cox regression with 10-fold cross-validation. Selected variables were entered into a multivariable Cox proportional hazards model. Internal validation repeated the complete model-development procedure in 1,000 bootstrap resamples. Model performance was assessed using Harrell's C-index, time-dependent area under the curve, calibration, Brier scores, and decision curve analysis.

**Results:**

Five predictors were retained: longer epilepsy duration, absence of a definitive structural MRI lesion, histopathological diagnosis, subtotal resection, and a high non-rapid eye movement interictal spike index (>38 spikes/h). The optimism-corrected C-index was 0.81 in the training cohort, and the C-index was 0.79 in the temporal validation cohort. The 1-year and 2-year time-dependent AUCs were 0.82 and 0.81 in the training cohort and 0.80 and 0.78 in the temporal validation cohort, respectively. Calibration was generally acceptable, and decision curve analysis suggested potential net benefit across the evaluated threshold range.

**Conclusions:**

The nomogram showed useful performance for estimating postoperative seizure-free survival in this single-center cohort. Because histopathological diagnosis and resection completeness are required, the model is intended for early postoperative risk stratification rather than preoperative candidate selection or presurgical counseling. Independent multicenter validation and prospective evaluation of clinical impact are required before routine implementation.

## 1 Introduction

Epilepsy is one of the most prevalent chronic neurological conditions globally, disproportionately affecting the pediatric population. Despite the continuous advancement and widespread availability of novel antiseizure medications, epidemiological studies consistently indicate that approximately 20% to 30% of pediatric patients fail to achieve sustained seizure control, subsequently developing drug-resistant epilepsy (1). In young children, the persistence of medically refractory seizures is particularly devastating. The rapidly developing pediatric brain is highly vulnerable to the deleterious neurobiological effects of recurrent, uncontrolled epileptiform networks and the cognitive dampening inevitably associated with high-dose polytherapy (1). Consequently, pediatric drug-resistant epilepsy is closely linked to progressive neurocognitive impairment, severe developmental delay, psychiatric comorbidities, and a significantly diminished overall quality of life for both the patients and their caregivers (2).

For children grappling with drug-resistant epilepsy, resective epilepsy surgery, specifically focal or lobar resection, has emerged as a highly efficacious and potentially curative intervention. Extensive clinical evidence demonstrates that successful surgical resection can drastically reduce or completely eliminate seizure burden, thereby offering a critical window for neurodevelopmental catch-up and fundamentally improving long-term psychosocial outcomes (2). However, achieving optimal surgical success remains a formidable clinical challenge that is heavily reliant on the precise presurgical localization of the epileptogenic zone. Despite rigorous presurgical evaluations involving high-resolution magnetic resonance imaging (MRI) and long-term scalp electroencephalography (EEG), approximately 30% to 40% of pediatric patients still experience residual seizures or late seizure recurrence following resective surgery (3, 4). Recent studies highlight that residual postoperative seizures profoundly negate the intended therapeutic benefits of surgery, leading to persistent morbidity and a marked deterioration in health-related quality of life (5). Therefore, accurate estimation of postoperative seizure-free survival may support early postoperative risk stratification, individualized follow-up and surveillance planning, and balanced communication with families regarding the risk of seizure recurrence.

Previous studies have identified several factors associated with seizure outcome after pediatric epilepsy surgery, including epilepsy duration, structural MRI findings, histopathological etiology, and completeness of resection (6). Although prediction models have been developed to combine these factors, their clinical application remains limited by restricted validation, inconsistent incorporation of electrophysiological information, and uncertainty regarding their performance across different patient populations (7). A clinically interpretable model integrating routinely available clinical, surgical, pathological, and EEG information may therefore improve individualized postoperative prognostic assessment.

The present study aimed to develop and temporally validate a multivariable prognostic nomogram for estimating 1-year and 2-year seizure-free survival after focal or lobar resection in children with drug-resistant epilepsy. The model incorporated clinical, imaging, surgical, histopathological, and semi-quantitative scalp EEG features and was intended to support postoperative risk stratification after the completeness of resection and final histopathological diagnosis became available.

## 2 Methods

### 2.1 Study design

This single-center retrospective observational cohort study was conducted to develop and temporally validate a predictive nomogram for seizure-free survival in pediatric patients with drug-resistant epilepsy undergoing focal or lobar resection. The study protocol and reporting strictly adhered to the Transparent Reporting of a Multivariable Prediction Model for Individual Prognosis or Diagnosis (TRIPOD) guidelines and the Strengthening the Reporting of Observational Studies in Epidemiology (STROBE) statement. Clinical, surgical, and electrophysiological data were consecutively retrieved from the institutional electronic medical record (EMR) system for patients treated between January 1, 2015, and December 31, 2024. A temporal validation strategy was implemented: patients enrolled from January 2015 to December 2021 constituted the training cohort for model development and internal validation, while those enrolled from January 2022 to December 2024 served as the independent temporal validation cohort. The study was approved by the Ethics Committee of Xinhua Hospital Affiliated to Shanghai Jiaotong University School of Medicine (Approval No.: XHEC-C-2025-182-1), and the requirement for written informed consent was waived by the Ethics Committee because of the retrospective nature of the study and the use of de-identified data, in accordance with the Declaration of Helsinki. All sensitive patient information was handled with strict confidentiality throughout the data extraction and analysis phases.

### 2.2 Study population

The study cohort was identified through a systematic query of the institutional EMR system and the epilepsy surgery database. All pediatric patients aged 1–18 years who underwent epilepsy surgery between January 1, 2015, and December 31, 2024, were initially screened. Potential participants underwent a three-stage validation process: (1) automated data extraction by the clinical data warehouse based on baseline demographic and surgical codes; (2) manual retrospective review of medical, surgical, and electrophysiological records by two independent epileptologists using standardized electronic case report forms; and (3) final adjudication by a senior neurosurgeon and an epileptologist in cases of diagnostic, surgical, or eligibility uncertainty. Discrepancies were resolved through consensus discussion.

Inclusion criteria comprised all of the following: (1) Confirmed diagnosis of drug-resistant epilepsy according to the International League Against Epilepsy (ILAE) consensus (8); (2) Age between 1 and 18 years at the time of surgery; (3) Underwent focal or lobar resection guided by comprehensive presurgical evaluation and multimodal imaging (9). Patients who underwent invasive intracranial EEG monitoring, including stereo-electroencephalography (SEEG), as part of the routine presurgical evaluation were not excluded and remained eligible provided that they subsequently underwent focal or lobar resection and fulfilled all other eligibility criteria; (4) Availability of complete presurgical baseline clinical data and standard long-term scalp electroencephalogram (EEG) records, specifically including at least a continuous 24-hour recording performed within 6 months prior to surgery, to allow for the extraction of standardized NREM sleep and wakefulness periods; (5) A minimum postoperative follow-up duration of 1 year, with clearly documented time-to-seizure recurrence (with any subsequent reoperation for epilepsy management during the follow-up period strictly defined as a recurrence event rather than censored data) or continuous seizure freedom to facilitate survival analysis. Exclusion criteria encompassed any of the following: (1) History of previous cranial surgery; (2) Underwent hemispherotomy, palliative procedures (e.g., vagus nerve stimulation, corpus callosotomy), or thermal ablation therapies; (3) Presence of severe progressive neurodegenerative or metabolic diseases; (4) Missing core survival outcome data (e.g., exact time of recurrence) or lack of definitive postoperative histopathological evaluation; (5) Missing data exceeding 20% for any candidate predictor, precluding reliable estimation through multiple imputation. A clinically documented decision not to perform a diagnostic examination was not considered missing data when the nonperformance itself constituted an observable presurgical evaluation category.

### 2.3 Data collection and outcome definition

Clinical and surgical data extraction was conducted through a double-blinded retrospective review of the electronic medical records. Candidate predictor variables were systematically collected based on established clinical relevance. Demographic and historical variables included age at seizure onset, age at the time of surgery, epilepsy duration, and a history of febrile seizures. Baseline neurocognitive status (defined as the presence or absence of developmental delay documented by a pediatric neurologist or neuropsychologist) and presurgical neuroimaging characteristics were also incorporated. Specifically, we documented the presence of a definitive structural lesion on magnetic resonance imaging (MRI) and the concordance of hypometabolism on fluorodeoxyglucose-positron emission tomography (FDG-PET) with the presumed epileptogenic zone. FDG-PET findings were classified as concordant, discordant, or not performed. The “not performed” category was retained as an observed clinical category and was not multiply imputed because FDG-PET was selectively omitted when the multidisciplinary team considered the structural MRI and other noninvasive findings sufficiently concordant for surgical planning. Thus, nonperformance could reflect the presurgical decision pathway rather than an unobserved PET result.

Invasive EEG monitoring was performed selectively when noninvasive presurgical investigations did not provide sufficiently concordant localization of the presumed epileptogenic zone. When SEEG was undertaken, its findings were reviewed by the multidisciplinary epilepsy surgery team and were used clinically to refine localization of the seizure-onset zone, assess its relationship to eloquent cortex, and determine the planned resection boundaries. However, SEEG status and individual invasive EEG findings were not included among the candidate predictors because invasive monitoring was used only in a clinically selected subgroup, its indications and electrode implantation schemes differed according to the presurgical hypothesis, and the retrospective records did not provide sufficiently standardized quantitative SEEG variables across the entire cohort. The final model therefore incorporated routinely available clinical, imaging, surgical, histopathological, and scalp EEG variables that could be consistently ascertained in all eligible patients.

Epilepsy-specific variables detailed the baseline seizure frequency (calculated as the average monthly seizure frequency over the 3 months immediately preceding the surgical evaluation), predominant seizure type, and the total number of concurrent antiepileptic drugs (ASMs) utilized at the time of surgical evaluation. Postoperative histopathological diagnoses were classified according to ILAE (10, 11) and WHO (12) criteria. Completeness of resection was determined via early postoperative MRI (within 48 h), distinguishing between gross total and subtotal resection.

Semi-quantitative EEG features were independently extracted by two board-certified clinical neurophysiologists blinded to surgical outcomes and histopathology. From the preoperative continuous EEG monitoring, one artifact-minimized two-hour epoch of non-rapid eye movement (NREM) sleep, including stages N2 and N3, and one two-hour epoch of wakefulness were selected for analysis. Visual interpretation and semi-quantitative assessment were performed using the standardized terminology of the International Federation of Clinical Neurophysiology revised glossary (13). The extracted features included background dominant rhythm frequency, amplitude distribution, spatial distribution of discharges classified as focal, multifocal, or diffuse, and ictal onset pattern.

The NREM interictal spike index was used as a study-specific semi-quantitative measure of interictal epileptiform discharge burden rather than as an established clinical scale with a universally accepted cutoff. Eligible spikes and sharp-wave discharges were counted during the standardized NREM epoch and expressed as the average number of discharges per analyzable hour. Components occurring within the same spike-and-wave complex were counted as a single discharge, whereas discharges recorded during electrographic seizures or within clearly artifactual segments were excluded. NREM sleep was selected for primary spike quantification because it facilitates the expression of interictal epileptiform discharges and provides a clinically accessible and relatively sensitive recording state.

The cutoff of >38 spikes/h was derived from the upper tertile of the spike-index distribution in the training cohort and was therefore data-driven rather than based on an established clinical threshold. Patients were classified as having a high (>38 spikes/h) or low/moderate (≤38 spikes/h) spike index, and the same fixed cutoff was applied to the temporal validation cohort. EEG features were independently reviewed by two board-certified clinical neurophysiologists who were blinded to postoperative outcomes. Disagreements were resolved by a third senior epileptologist. Inter-rater agreement for categorical EEG features was assessed using Cohen's kappa, whereas reproducibility of continuous spike counts was assessed using the intraclass correlation coefficient.

A total of 18 prespecified candidate predictors were evaluated for model development: sex, age at seizure onset, age at surgery, epilepsy duration, history of febrile seizures, developmental delay, baseline monthly seizure frequency, predominant seizure type, number of concurrent antiseizure medications, presence of a definitive structural lesion on MRI, FDG-PET concordance, background dominant rhythm frequency, amplitude distribution, spatial distribution of epileptiform discharges, ictal onset pattern, NREM interictal spike index, histopathological diagnosis, and completeness of resection. These variables were selected before model fitting based on their clinical availability, previously reported prognostic relevance, and consistent ascertainment within the institutional epilepsy surgery database.

The primary outcome was seizure-free survival, defined as the time to seizure recurrence following surgery. Postoperative outcomes were categorized using the Engel Epilepsy Surgery Outcome Scale (14). An event was defined as the first occurrence of an Engel Class II-IV outcome (indicating any seizure recurrence, including disabling auras) or the requirement for secondary epilepsy-directed cranial surgery. The precise time of recurrence was calculated in months from the date of the primary surgery to the date of the aforementioned event. Patients who maintained an Engel Class I outcome throughout the two-year follow-up, or those lost to follow-up without a documented recurrence, were right-censored at their last clinical contact.

### 2.4 Sample size estimation

To ensure adequate statistical power and prevent model overfitting in the Cox proportional hazards regression analysis, we prospectively constrained the final multivariable model to encompass a maximum of 4–6 core predictors out of 15–20 initial candidate variables. According to the modern methodological criteria for developing and validating clinical prediction models, maintaining an events-per-variable (EPV) ratio of at least 10 is fundamental to deriving robust hazard estimates and preventing optimism in survival models (15). Based on extensive multicenter pediatric epilepsy surgery cohorts, the estimated postoperative seizure recurrence rate for focal resections is consistently reported to be approximately 35% (corresponding to a 65% seizure-free survival rate) within the initial years of follow-up (16). To comfortably satisfy the EPV ≥ 10 threshold for a maximum of 6 multivariable predictors, a minimum of 60 recurrence events was mandated within the training cohort. Given the 35% estimated event rate, this necessitated a training cohort size of at least 171 patients. Incorporating our predefined 7:3 temporal split ratio for model derivation and validation, the study consecutively enrolled a total cohort of 250 pediatric patients. This specific configuration yielded 175 patients (containing 62 actual recurrence events) in the training cohort and 75 patients in the temporal validation cohort.

### 2.5 Statistical analysis

Missingness was assessed for each candidate predictor before model development. Missing values occurred for age at seizure onset in 8 of 256 patients (3.1%), history of febrile seizures in 15 patients (5.9%), and developmental delay in 11 patients (4.3%). No data were missing for the primary outcome, follow-up time, MRI lesional status, histopathological diagnosis, completeness of resection, or interictal spike index. Multiple imputation by chained equations was performed under a missing-at-random assumption to generate five imputed datasets. Predictive mean matching was used for age at seizure onset, and binary logistic regression was used for history of febrile seizures and developmental delay. The imputation model included all candidate predictors, temporal cohort, recurrence status, and the Nelson–Aalen cumulative hazard estimate. Twenty iterations were run per dataset, and convergence was assessed using trace plots and comparison of observed and imputed distributions. Cox regression estimates were pooled using Rubin's rules. FDG-PET status was treated as a three-level observed variable—concordant, discordant, or not performed—and was not imputed.

Continuous variables were summarized as mean ± standard deviation or median with interquartile range, as appropriate, and categorical variables as frequencies and percentages. Training and temporal validation cohorts were compared using independent-samples t-tests, Mann–Whitney U tests, Pearson's *χ*^2^ tests, or Fisher's exact tests. All tests were two-sided, with *p* < 0.05 considered statistically significant.

Within the training cohort, the 18 prespecified candidate predictors described above were entered into a least absolute shrinkage and selection operator (LASSO) penalized Cox regression with 10-fold cross-validation for feature selection and dimensionality reduction. The penalty parameter was selected using lambda.1se. Variance inflation factors were used to assess multicollinearity, with values <5 considered acceptable. Selected predictors were entered into a multivariable Cox proportional hazards model to estimate hazard ratios and 95% confidence intervals. The proportional hazards assumption was evaluated using global and covariate-specific tests based on scaled Schoenfeld residuals and graphical inspection. A nomogram was constructed to estimate 1-year and 2-year seizure-free survival probabilities.

Internal validation was performed using 1,000 bootstrap resamples. In each resample, the prespecified modeling procedure was applied, including LASSO penalized Cox regression, 10-fold cross-validation for selection of lambda.1se, predictor selection, and refitting of the multivariable Cox model. Optimism was estimated from the average difference between bootstrap-sample and original-sample performance. Temporal validation was performed by applying the complete model equation, including the original regression coefficients and baseline survival estimates, without refitting or recalibration.

A nomogram was constructed from the final Cox model to estimate individualized 1-year and 2-year seizure-free survival probabilities. Exact point allocations for each predictor and the mapping of total points to predicted survival probabilities are provided in Supplementary Tables S1, S2, respectively.

Discrimination was evaluated using Harrell's concordance index and time-dependent receiver operating characteristic curves at 1 and 2 years. The numbers at risk and cumulative recurrence and censoring counts were calculated at both time points. Calibration was assessed by comparing model-predicted and Kaplan–Meier-estimated probabilities and by the Greenwood–Nam–D’Agostino test. Prediction error was quantified using Brier scores. The 95% confidence intervals for time-dependent AUCs and Brier scores were estimated using 1,000 bootstrap resamples. Decision curve analysis was used to assess net clinical benefit. All analyses were conducted using R software, version 4.5.1.

## 3 Results

### 3.1 Baseline characteristics of the study population

**Figure 1:**
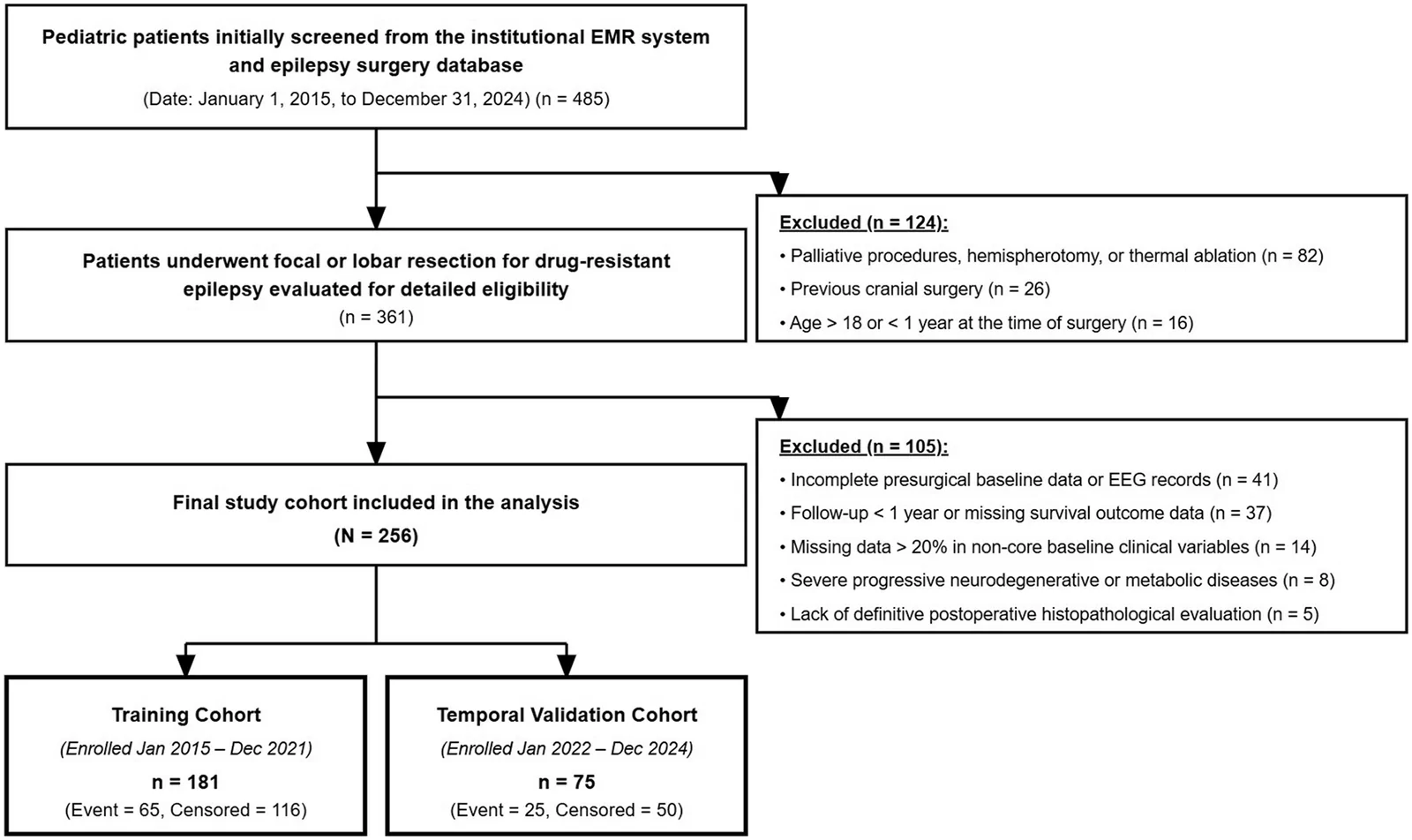
Flowchart of patient screening, inclusion, and exclusion process.

A total of 256 pediatric patients were included (Figure 1) and temporally divided into a training cohort (*n* = 181; 2015–2021) and a temporal validation cohort (*n* = 75; 2022–2024). Missing values were limited to age at seizure onset (8/256, 3.1%), history of febrile seizures (15/256, 5.9%), and developmental delay (11/256, 4.3%). FDG-PET was not performed in 60 patients (23.4%); these observations were retained as a separate clinical category rather than treated as missing values. Inter-rater agreement was high for the categorical EEG features (Cohen's *κ* = 0.84), and reproducibility of the continuous NREM interictal spike counts was also high (intraclass correlation coefficient = 0.91, 95% CI: 0.88–0.93). Baseline characteristics are detailed in Table 1. Overall, the median baseline seizure frequency was 14 (IQR, 6–27) episodes per month, and structural MRI lesions were present in 82.0% of patients. Focal cortical dysplasia was the predominant etiology (45.7%), followed by CNS tumors (28.9%). Median follow-up duration was significantly longer in the training cohort than in the temporal validation cohort (60 vs. 24 months, *p* < 0.001). Recurrence events occurred in 65 (35.9%) patients in the training cohort and 25 (33.3%) in the temporal validation cohort. The numbers of patients remaining at risk at 1 and 2 years were 160 and 139, respectively, in the training cohort and 67 and 39, respectively, in the temporal validation cohort. By 2 years, 6 patients in the training cohort and 21 patients in the temporal validation cohort had been censored without documented seizure recurrence (Supplementary Table S3). Baseline covariates, including neurocognitive status, completeness of resection, and standardized spike index, were comparable between the two cohorts (all *p* > 0.05; Table 1).

**Table 1:** Baseline demographic, clinical, electrophysiological, and surgical characteristics of the training and temporal validation cohorts.

Characteristics	Overall (*N* = 256)	Training Cohort (*n* = 181)	Temporal validation cohort (*n* = 75)	Test Statistic	*P*-value
Demographic and Historical Features					
Male sex, *n* (%)	136 (53.1)	98 (54.1)	38 (50.7)	*χ*2 = 0.25	0.615
Age at seizure onset (years) (*n* = 248), Mean ± SD	4.6 ± 3.1	4.5 ± 2.9	4.8 ± 3.4	t = −0.71	0.476
Age at surgery (years), Mean ± SD	10.1 ± 3.8	10.2 ± 3.7	9.8 ± 4.1	t = 0.74	0.460
Epilepsy duration (years), Mean ± SD	5.5 ± 3.0	5.7 ± 2.8	5.0 ± 3.3	t = 1.69	0.092
History of febrile seizures (n = 241), *n* (%)	65 (27.0)	46 (27.1)	19 (26.8)	χ2 = 0.01	0.957
Developmental delay (n = 245), *n* (%)	106 (43.3)	73 (42.2)	33 (45.8)	χ2 = 0.29	0.593
Epilepsy-Specific Features					
Baseline seizure frequency (per month), Median (IQR)	14 (6–27)	15 (6–28)	12 (5–25)	Z = 1.10	0.269
Predominant seizure type, *n* (%)				χ2 = 0.15	0.700
Focal aware	91 (35.5)	63 (34.8)	28 (37.3)		
Focal impaired awareness/Bilateral	165 (64.5)	118 (65.2)	47 (62.7)		
Concurrent ASMs at surgery, Median (IQR)	3 (2–4)	3 (2–4)	3 (2–4)	Z = 0.41	0.686
Neuroimaging Features					
Definitive structural lesion on MRI, *n* (%)	210 (82.0)	147 (81.2)	63 (84.0)	χ2 = 0.29	0.591
FDG-PET concordance, *n* (%)				χ2 = 0.47	0.790
Concordant	156 (60.9)	112 (61.9)	44 (58.7)		
Discordant	40 (15.6)	27 (14.9)	13 (17.3)		
Not performed	60 (23.4)	42 (23.2)	18 (24.0)		
Semi-Quantitative EEG Features					
Background dominant rhythm (Hz), Mean ± SD	7.8 ± 1.4	7.7 ± 1.4	7.9 ± 1.5	t = −0.99	0.325
Amplitude distribution, *n* (%)				χ2 = 0.13	0.724
Normal symmetry	163 (63.7)	114 (63.0)	49 (65.3)		
Asymmetric/Abnormal	93 (36.3)	67 (37.0)	26 (34.7)		
Spatial distribution of discharges, *n* (%)				χ2 = 0.32	0.854
Focal	137 (53.5)	96 (53.0)	41 (54.7)		
Multifocal	76 (29.7)	55 (30.4)	21 (28.0)		
Diffuse	43 (16.8)	30 (16.6)	13 (17.3)		
Ictal onset pattern, *n* (%)				χ2 = 0.19	0.665
Focal	183 (71.5)	128 (70.7)	55 (73.3)		
Regional/Non-lateralizing	73 (28.5)	53 (29.3)	20 (26.7)		
Interictal Spike Index, *n* (%)				χ2 = 0.08	0.784
Low/Moderate (≤ 38 spikes/h)	171 (66.8)	120 (66.3)	51 (68.0)		
High (> 38 spikes/h)	85 (33.2)	61 (33.7)	24 (32.0)		
Surgical and Pathological Features					
Histopathological diagnosis, *n* (%)				χ2 = 0.21	0.900
Focal cortical dysplasia (FCD)	117 (45.7)	82 (45.3)	35 (46.7)		
CNS tumors	74 (28.9)	54 (29.8)	20 (26.7)		
Others (incl. HS and gliosis/cavernoma)	65 (25.4)	45 (24.9)	20 (26.7)		
Completeness of resection, *n* (%)				χ2 = 0.63	0.428
Gross total resection	193 (75.4)	134 (74.0)	59 (78.7)		
Subtotal resection	63 (24.6)	47 (26.0)	16 (21.3)		
Outcome					
Follow-up duration (months), Median (IQR)	48 (24–72)	60 (36–84)	24 (18–36)	Z = 6.45	< 0.001
Seizure recurrence event, *n* (%)	90 (35.2)	65 (35.9)	25 (33.3)	χ2 = 0.15	0.697

Table 1 summarizes the original observed data before multiple imputation. Missing values occurred for age at seizure onset (8/256, 3.1%), history of febrile seizures (15/256, 5.9%), and developmental delay (11/256, 4.3%). These variables were multiply imputed using chained equations. The “not performed” level of FDG-PET represented a documented clinical decision not to obtain PET imaging and was retained as an observed category rather than treated as missing data. No values were missing for the primary outcome or the five predictors retained in the final Cox model. SD, standard deviation; IQR, interquartile range; ASMs, antiepileptic seizure medications; MRI, magnetic resonance imaging; FDG-PET, fluorodeoxyglucose-positron emission tomography; EEG, electroencephalogram; CNS, central nervous system; HS, hippocampal sclerosis. Continuous variables following a normal distribution were analyzed using independent samples t-tests (reported with t statistics), whereas skewed data were analyzed using Mann–Whitney U tests (reported with Z statistics). Categorical variables were assessed using Pearson's Chi-square tests (reported with χ2 statistics) or Fisher's exact tests as appropriate. A two-sided *p*-value < 0.05 was considered statistically significant. Standardized time windows for EEG features were derived from a continuous two-hour non-rapid eye movement (NREM) sleep epoch and a two-hour wakefulness epoch. The interictal spike index was dichotomized into high and low/moderate frequency cohorts utilizing the upper tertile threshold derived from the training cohort distribution.

### 3.2 Feature selection by LASSO regression

**Figure 2:**
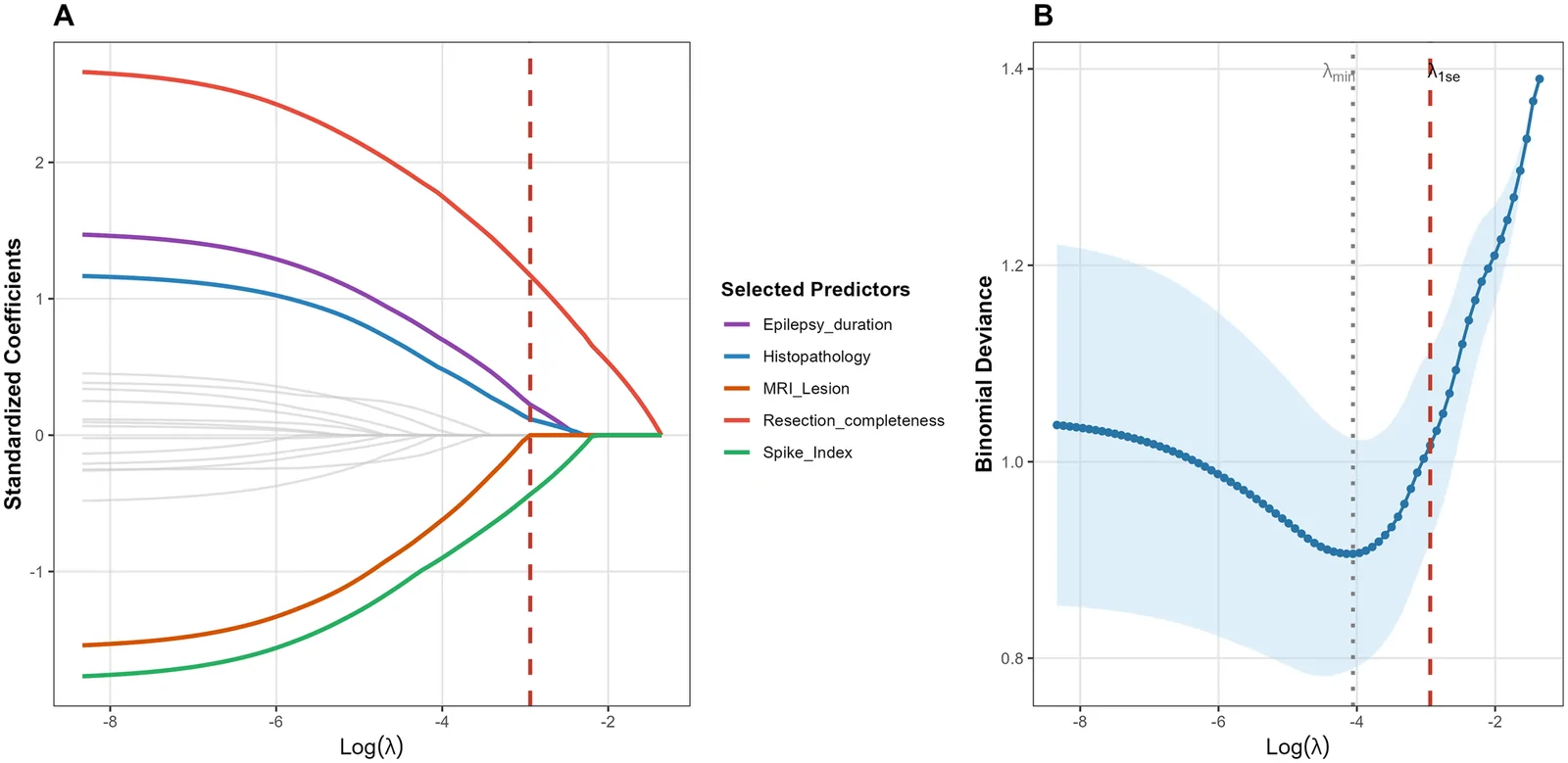
Feature selection using the least absolute shrinkage and selection operator (LASSO) penalized Cox regression model in the training cohort. **(A)** Coefficient profiles of the 18 prespecified clinical, neuroimaging, scalp EEG, surgical, and pathological candidate predictors listed in the Methods. Each curve represents a single variable, illustrating how its coefficient approaches zero as the penalty parameter (λ) increases. **(B)** Ten-fold cross-validation curve for tuning the penalty parameter in the LASSO model. The binomial deviance is plotted against log (λ). The two vertical dotted lines indicate the optimal values defined by the minimum criteria (lambda.min) and the 1-standard error criteria (lambda.1se), with the latter driving the selection of the 5 final variables.

LASSO penalized Cox regression with 10-fold cross-validation was applied to the 18 prespecified clinical, neuroimaging, scalp EEG, surgical, and pathological candidate predictors listed in the Methods in the training cohort (*n* = 181) (Figure 2A). Using the 1-standard error criterion (lambda.1se = 0.058) to promote model parsimony, 13 variables were shrunk to zero (Figure 2B). Five predictors with non-zero coefficients were retained: epilepsy duration, presence of a definitive structural lesion on MRI, histopathological diagnosis, completeness of resection, and NREM interictal spike index. These variables were subsequently entered into the multivariable Cox regression model.

### 3.3 Development of the predictive nomogram

**Figure 3:**
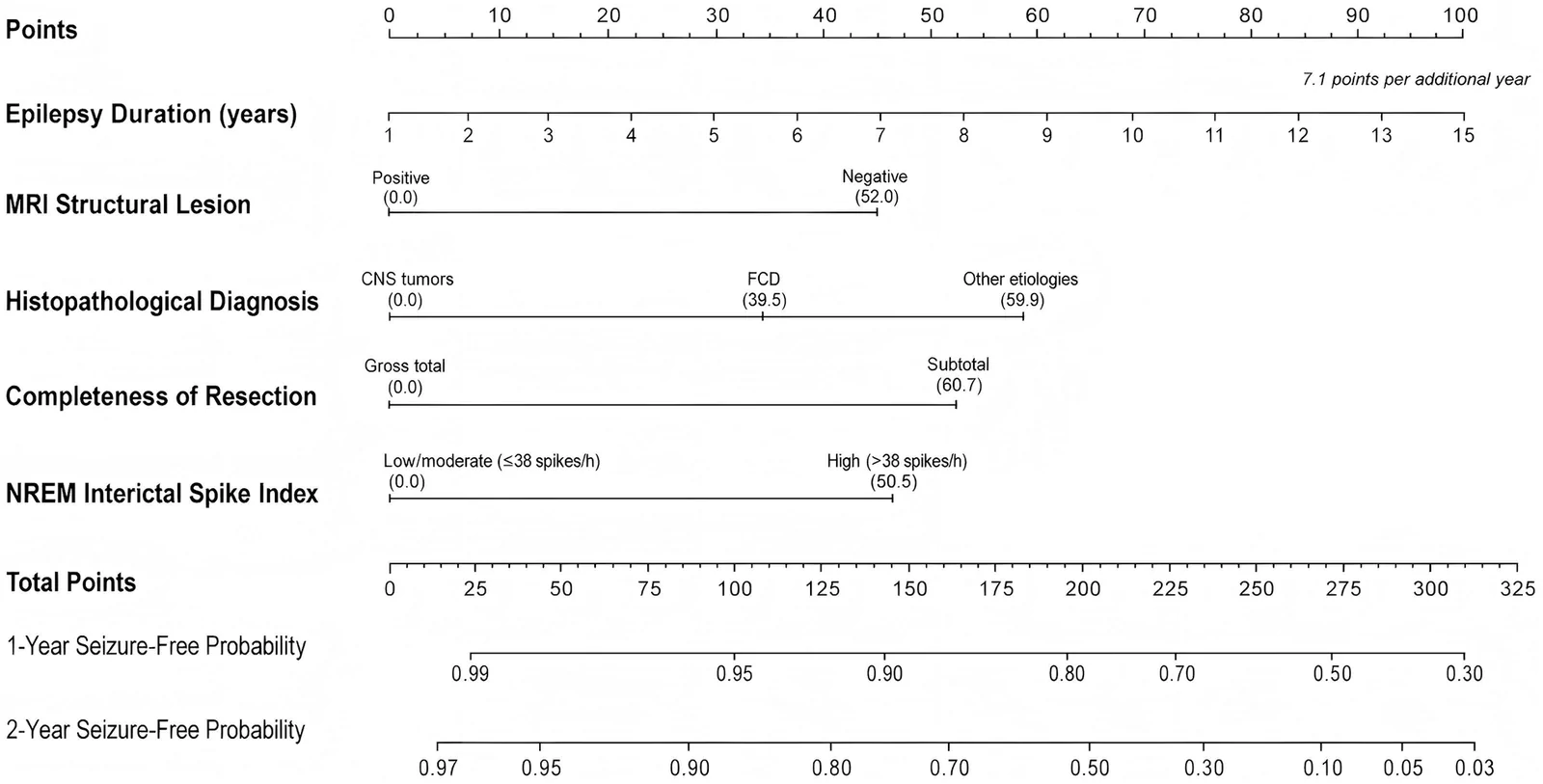
Nomogram for estimating 1-year and 2-year seizure-free survival probabilities after focal epilepsy surgery in children. For each predictor, a vertical line is drawn to the upper Points axis to determine the corresponding score. The individual scores are summed to obtain the Total Points, which are then projected onto the 1-year and 2-year seizure-free probability axes. Exact point allocations and selected total-point probability mappings are provided in Supplementary Tables S1, S2, respectively. MRI, magnetic resonance imaging; FCD, focal cortical dysplasia; NREM, non-rapid eye movement.

Multivariable Cox proportional hazards regression in the training cohort confirmed that all five LASSO-selected variables were independent predictors of postoperative seizure recurrence (Table 2). Specifically, longer epilepsy duration (HR = 1.11, 95% CI: 1.02–1.21, *p* = 0.012), negative MRI (HR = 2.15, 95% CI: 1.17–3.96, *p* = 0.014), subtotal resection (HR = 2.44, 95% CI: 1.51–3.94, *p* < 0.001), and a high interictal spike index (>38 spikes/h; HR = 2.10, 95% CI: 1.27–3.46, *p* = 0.004) were associated with increased recurrence hazard. Conversely, CNS tumors were associated with a lower hazard compared to FCD (HR = 0.56, 95% CI: 0.34–0.93, *p* = 0.026). A nomogram predicting 1-year and 2-year seizure-free survival probabilities was constructed based on the final Cox model (Figure 3). Exact point allocations for the five predictors and the corresponding mapping between total points and predicted seizure-free survival probabilities are presented in Supplementary Tables S1, S2.

**Table 2:** Multivariable Cox proportional hazards regression analysis of seizure recurrence in the training cohort.

Predictor	HR (95% CI)	*P*-value
Epilepsy duration, per 1-year increase	1.11 (1.02–1.21)	0.012
Definitive structural lesion on MRI		
Positive	Reference	—
Negative	2.15 (1.17–3.96)	0.014
Histopathological diagnosis		
Focal cortical dysplasia	Reference	—
CNS tumor	0.56 (0.34–0.93)	0.026
Other etiologies, including hippocampal sclerosis, gliosis, and cavernoma	1.35 (0.79–2.31)	0.275
Completeness of resection		
Gross total resection	Reference	—
Subtotal resection	2.44 (1.51–3.94)	<0.001
NREM interictal spike index		
Low/moderate, ≤38 spikes/h	Reference	—
High, >38 spikes/h	2.10 (1.27–3.46)	0.004

HR, hazard ratio; CI, confidence interval; MRI, magnetic resonance imaging; NREM, non-rapid eye movement. The event was postoperative seizure recurrence, defined as Engel Class II–IV or epilepsy-related reoperation. HR >1 indicates a higher recurrence hazard, whereas HR <1 indicates a lower recurrence hazard. All predictors satisfied the proportional hazards assumption based on scaled Schoenfeld residuals. The maximum variance inflation factor was <2.0.

### 3.4 Performance, validation, and clinical utility of the nomogram

**Figure 4:**
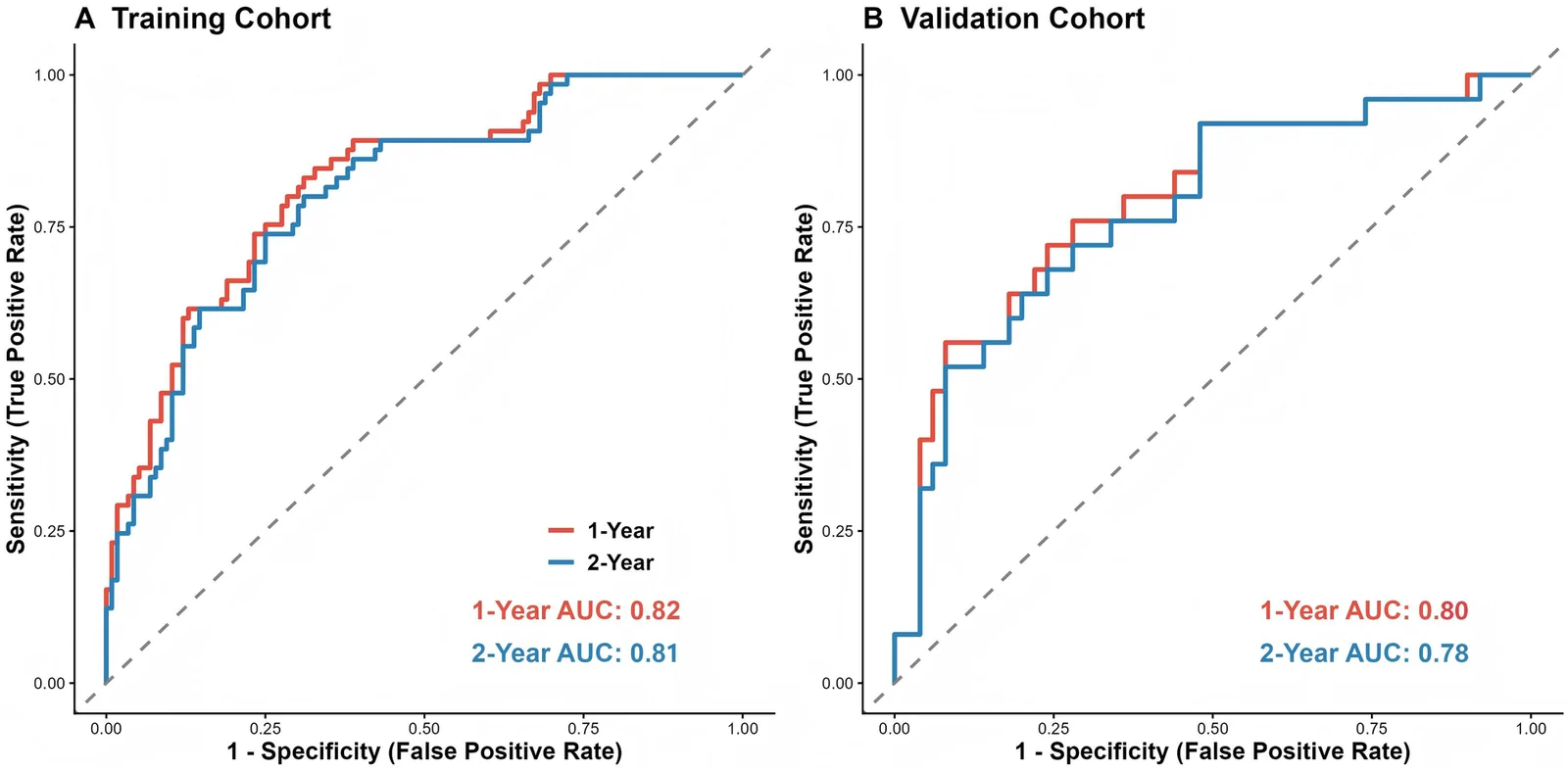
Time-dependent receiver operating characteristic (ROC) curves for the predictive nomogram. **(A)** 1-year and 2-year ROC curves with corresponding Area Under the Curve (AUC) values in the training cohort. **(B)** 1-year and 2-year ROC curves with corresponding AUC values in the temporal validation cohort.

**Figure 5:**
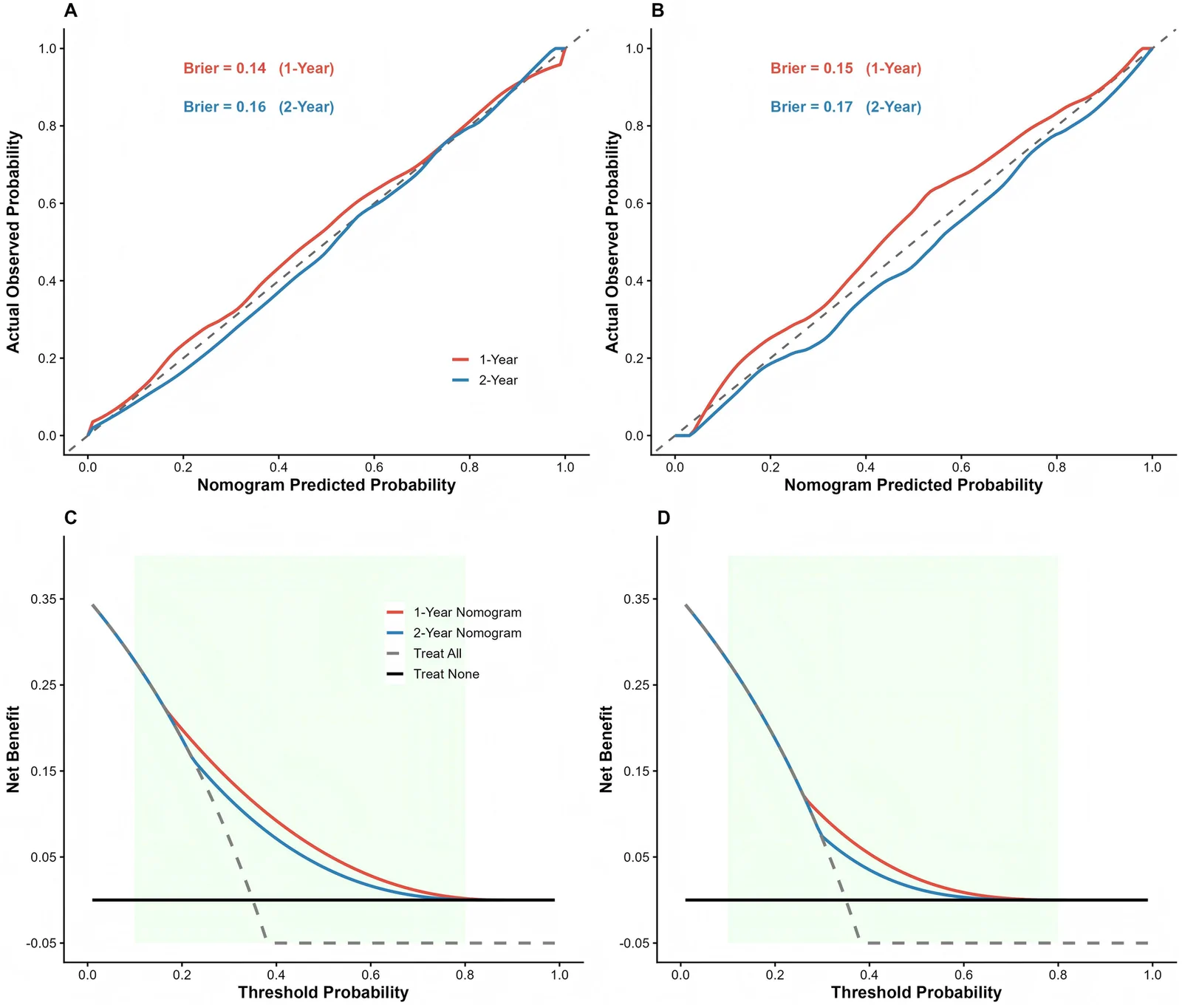
Calibration and clinical utility of the nomogram for predicting seizure-free survival. **(A,B)** Calibration curves for 1-year and 2-year survival probabilities in the training and validation cohorts, respectively. The dashed 45-degree line represents perfect prediction, while the solid lines represent the model's actual performance. **(C,D)** Decision curve analysis (DCA) for 1-year and 2-year survival predictions. The *y*-axis measures the net clinical benefit. The nomogram curves were generally above the “treat-all” and “treat-none” strategies across the evaluated threshold probability range, suggesting potential net clinical benefit under the assumptions of the decision curve analysis.

The nomogram yielded a bootstrap-corrected C-index of 0.81 (95% CI: 0.77–0.86) in the training cohort and 0.79 (95% CI: 0.71–0.86) in the temporal validation cohort. Time-dependent ROC analysis showed AUCs of 0.82 (95% CI: 0.77–0.87) and 0.81 (95% CI: 0.75–0.86) for 1-year and 2-year seizure-free survival, respectively, in the training cohort, and 0.80 (95% CI: 0.70–0.87) and 0.78 (95% CI: 0.68–0.86), respectively, in the temporal validation cohort (Figure 4). Calibration plots demonstrated close agreement between predicted and observed survival probabilities (Figures 5A,B). The Brier scores were 0.14 (95% CI: 0.11–0.17) at 1 year and 0.16 (95% CI: 0.12–0.19) at 2 years in the training cohort, and 0.15 (95% CI: 0.11–0.21) and 0.17 (95% CI: 0.12–0.23), respectively, in the temporal validation cohort. Greenwood–Nam–D’Agostino tests were nonsignificant at both time points in both cohorts (all *p* > 0.05). Finally, DCA indicated that the nomogram provided a higher net clinical benefit than both “treat-all” and “treat-none” strategies across a wide threshold probability range (10%–80%) in both cohorts (Figures 5C,D).

## 4 Discussion

In this single-center retrospective cohort study, we developed and temporally validated a multivariable nomogram for estimating 1-year and 2-year seizure-free survival after focal or lobar resection in children with drug-resistant epilepsy. The final model included epilepsy duration, structural MRI lesion status, histopathological diagnosis, completeness of resection, and the NREM interictal spike index. The model showed good discrimination and generally acceptable calibration in the training and temporal validation cohorts. Because histopathological diagnosis and postoperative assessment of resection completeness are required, the nomogram should be interpreted as an early postoperative risk-stratification tool rather than a preoperative instrument for candidate selection or presurgical counseling.

The intended users are pediatric epileptologists, epilepsy surgeons, and multidisciplinary epilepsy teams. The nomogram can be applied after final histopathological assessment and early postoperative MRI have established the pathological diagnosis and completeness of resection, typically during the first postoperative multidisciplinary review. Its purpose is to estimate the probability of remaining seizure-free over the subsequent 1 and 2 years and to support the planning of postoperative surveillance. Patients with higher predicted recurrence risk may warrant closer clinical follow-up, earlier postoperative EEG assessment, review of residual structural or electrophysiological abnormalities, and timely reassessment if seizures recur. The score may also provide contextual information when clinicians consider the timing of antiseizure medication reduction, but it should not independently determine medication withdrawal or prompt reoperation. Such decisions require consideration of postoperative seizure course, EEG findings, medication tolerability, lesion type, and family preferences.

Longer epilepsy duration was associated with a higher hazard of postoperative seizure recurrence. This association is consistent with previous observational studies reporting poorer seizure outcomes among patients with longer-standing epilepsy (17). Prolonged epileptic activity may be accompanied by expansion or reorganization of epileptogenic networks, which provides a biologically plausible explanation for the observed relationship (18, 19). However, the present study cannot establish that delaying surgery itself caused the poorer outcome. Children who underwent surgery later may have differed systematically from those treated earlier in terms of MRI-negative disease, multifocal or poorly localized epileptogenic activity, diagnostic complexity, referral pathways, access to specialized care, or initial surgical candidacy. Reverse causation is also possible because patients with less clearly resectable epileptogenic zones may require longer evaluation before surgery and may independently have less favorable outcomes. Epilepsy duration should therefore be regarded as a prognostic marker within this cohort rather than evidence that a specified reduction in time to surgery would necessarily improve seizure freedom. Nevertheless, timely referral for comprehensive evaluation remains clinically appropriate for children with drug-resistant epilepsy.

Structural MRI lesion status and completeness of resection were associated with postoperative seizure outcome. MRI-negative epilepsy poses a localization challenge because a discrete anatomical substrate is not readily identifiable, and these patients may require additional functional imaging or invasive EEG evaluation (20, 21). In this cohort, SEEG was used selectively in patients with complex or discordant noninvasive findings and informed localization and resection planning. Because SEEG utilization reflected the preceding clinical evaluation pathway and standardized invasive EEG variables were unavailable across the full cohort, the associations of MRI-negative status and subtotal resection should not be interpreted independently of case complexity or selection for invasive monitoring. Previous studies have consistently identified complete removal of the structural or electroclinically defined epileptogenic zone as an important correlate of seizure freedom (16). Advanced MRI post-processing, PET, and other multimodal techniques may improve localization in selected MRI-negative cases, although their incremental value depends on the clinical context and institutional workflow (22).

Histopathological diagnosis also contributed to risk stratification. Patients with central nervous system tumors had a lower recurrence hazard than those with focal cortical dysplasia. Many pediatric low-grade epilepsy-associated tumors have relatively discrete pathological and imaging boundaries, which can facilitate complete lesion removal (23). In contrast, FCD may extend beyond the MRI-visible abnormality, and the histological transition into surrounding cortex can be difficult to define intraoperatively (24). Large multicenter histopathological cohorts have similarly shown that postoperative seizure outcomes vary according to pathological substrate, with low-grade epilepsy-associated tumors generally having favorable outcomes (16).

FCD should not, however, be regarded as a uniform pathological entity. FCD type I is often subtler on MRI and may involve more diffuse cortical architectural abnormalities, whereas FCD type II, particularly type IIb, is often more conspicuous and spatially localized. These differences may affect lesion detection, delineation of the epileptogenic zone, resection completeness, and postoperative outcome. Previous comparative studies have reported clinically and radiologically distinct patterns between FCD types I and II, although seizure-outcome differences have not been uniform across series (25).

A reliable subtype-specific analysis was not performed in the present study because subtype-level classification was not consistently available in a standardized format across the entire 2015–2024 study period, and the number of recurrence events within individual Ia, Ib, IIa, and IIb strata was insufficient for stable multivariable estimation. FCD was therefore retained as a single prespecified pathological category. This grouping may have obscured prognostic heterogeneity between more diffuse type I lesions and more circumscribed type II lesions. Future multicenter studies with central neuropathological review should evaluate whether FCD subtype provides incremental prognostic information beyond MRI lesion status and resection completeness.

The NREM interictal spike index provided a semi-quantitative estimate of interictal epileptiform discharge burden. Interictal spike frequency has been investigated as a marker of cortical irritability and epileptogenic network activity (26, 27). NREM sleep facilitates the expression of interictal discharges through increased neuronal synchronization and therefore offers a sensitive state for evaluating discharge burden. Studies using magnetoencephalography and intracranial recordings suggest that frequent or spatially extensive interictal discharges may indicate a broader epileptogenic network that is less amenable to localized resection (27, 28).

The measure used in this study—visually counted discharges per analyzable hour during a standardized two-hour NREM epoch—was study-specific and should not be interpreted as a universally established clinical scale. The cutoff of >38 spikes/h was derived from the upper tertile of the training cohort and represents a data-driven risk-stratification threshold rather than a validated biological boundary. A higher spike index was associated with recurrence, but scalp-recorded discharge burden can also vary with sleep stage, recording quality, antiseizure medication exposure, lesion location, and electrode sensitivity. The association is therefore prognostic rather than causal. Routine scalp EEG and the standardized sampling method may make the index feasible in specialist centers, but reproducible use requires consistent sleep-stage selection, artifact exclusion, and discharge-identification criteria. More extensive presurgical electrophysiological mapping, including high-frequency oscillation analysis or source-connectivity assessment, may still be required for patients with complex or widespread epileptogenic networks (27, 29).

The present study also addresses several methodological limitations discussed in previous prediction research. Rather than treating seizure outcome solely as a fixed binary endpoint, the Cox model incorporated the timing of seizure recurrence and generated estimates at clinically relevant 1-year and 2-year time points. Candidate predictors were selected using penalized regression, and the complete model-development procedure was included in bootstrap internal validation. The later temporal cohort was evaluated without model refitting or recalibration. In addition, calibration, prediction error, and decision curve analysis were considered alongside discrimination, providing a broader assessment than the AUC alone (7). These features improve the transparency of model evaluation but do not eliminate the limitations of retrospective model development.

The temporal validation results indicate that the model retained useful predictive performance in a later cohort from the same institution. However, temporal validation is not equivalent to external validation because both cohorts shared the same referral setting, presurgical evaluation protocols, EEG acquisition procedures, surgical team, and pathological practices. Decision curve analysis suggested potential net benefit across the evaluated threshold range, but it does not demonstrate that use of the nomogram improves patient outcomes in practice. Clinical utility will require prospective impact evaluation showing that score-informed follow-up or management decisions provide benefit beyond standard multidisciplinary care.

Several limitations should be considered. First, the retrospective observational design creates risks of selection bias, unmeasured confounding, and reverse causation and precludes causal interpretation of the predictors. Second, the single-center setting limits transportability despite the use of a later temporal validation cohort. Independent multicenter validation is required before routine implementation. Third, missing values for age at seizure onset, febrile seizure history, and developmental status were imputed under a missing-at-random assumption that cannot be verified. FDG-PET nonperformance was retained as an observed category because it reflected the clinical evaluation pathway, but residual confounding related to selective imaging remains possible. SEEG was also selectively used in more complex cases, and standardized invasive EEG features were not modeled. Fourth, grouping all FCD subtypes into one category may have concealed differences between type I and type II lesions. Fifth, although the spike index showed good inter-reader reproducibility under the study protocol, visual counting remains dependent on expertise and recording quality. The >38 spikes/h cutoff was data-driven and may not transfer to populations with different EEG protocols or case mixes. Finally, the model did not include automated EEG biomarkers, advanced functional connectivity measures, or molecular genetic data. Future prospective multicenter studies should externally validate the locked model, examine FCD subtypes with standardized neuropathological review, and determine whether continuous or automated EEG measures improve prediction beyond routinely available variables.

## 5 Conclusion

This study developed and temporally validated a nomogram for estimating 1-year and 2-year seizure-free survival after focal or lobar epilepsy surgery in children. The model combined epilepsy duration, structural MRI lesion status, histopathological diagnosis, completeness of resection, and the NREM interictal spike index. Because two predictors become available only after surgery, the nomogram is intended for early postoperative risk stratification rather than preoperative candidate selection. It may complement multidisciplinary assessment when planning follow-up and postoperative evaluation, but it should not independently determine antiseizure medication withdrawal or further intervention. Independent multicenter validation and prospective assessment of clinical impact are required before routine implementation.

## Data Availability

The raw data supporting the conclusions of this article will be made available by the authors, without undue reservation.
