# Supplementary material for: Development and temporal validation of a postoperative nomogram based on clinical, surgical, histopathological, and semi-quantitative EEG features for predicting seizure-free survival in children after focal epilepsy surgery
Source: Front Pediatr. 2026 Aug 28;14:1869297. doi: 10.3389/fped.2026.1869297 (PMC13561969; doi:10.3389/fped.2026.1869297)
Supplement: Supplementary file 1 [file Table1.docx]

**Supplementary Table S1.** Exact point allocation for predictors included in the nomogram

| **Predictor** | **Category or value** | **Assigned points** |
| --- | --- | --- |
| Epilepsy duration, years | 1 | 0.0 |
|  | Each additional year | +7.1 |
|  | 5 | 28.6 |
|  | 10 | 64.3 |
|  | 15 | 100.0 |
| MRI structural lesion | Positive | 0.0 |
|  | Negative | 52.0 |
| Histopathological diagnosis | CNS tumors | 0.0 |
|  | Focal cortical dysplasia | 39.5 |
|  | Other etiologies | 59.9 |
| Completeness of resection | Gross total | 0.0 |
|  | Subtotal | 60.7 |
| NREM interictal spike index | Low/moderate, ≤38 spikes/h | 0.0 |
|  | High, >38 spikes/h | 50.5 |

Note: Points for epilepsy duration were calculated as 7.14 × (duration in years − 1), with the observed range restricted to 1–15 years. Point values were derived from the unrounded regression coefficients of the final Cox model. The maximum contribution of epilepsy duration was scaled to 100 points. Within each categorical predictor, the category associated with the lowest recurrence hazard was assigned 0 points, and the remaining categories were assigned points according to their coefficient differences relative to that category. Accordingly, CNS tumors were assigned 0 points for histopathological diagnosis, although focal cortical dysplasia was the reference category in the Cox regression table. NREM, non-rapid eye movement; MRI, magnetic resonance imaging.

**Supplementary Table S2.** Mapping of total nomogram points to predicted 1-year and 2-year seizure-free survival probabilities

| **Total points** | **Predicted 1-year seizure-free probability** | **Predicted 2-year seizure-free probability** |
| --- | --- | --- |
| 0 | 0.990 | 0.970 |
| 25 | 0.986 | 0.957 |
| 50 | 0.979 | 0.938 |
| 75 | 0.970 | 0.912 |
| 100 | 0.957 | 0.876 |
| 125 | 0.938 | 0.826 |
| 150 | 0.913 | 0.759 |
| 175 | 0.876 | 0.671 |
| 200 | 0.827 | 0.562 |
| 225 | 0.760 | 0.434 |
| 250 | 0.673 | 0.301 |
| 275 | 0.563 | 0.178 |
| 300 | 0.437 | 0.082 |
| 325 | 0.303 | 0.027 |

Note: Predicted seizure-free survival probabilities were calculated from the locked final Cox model using the unrounded regression coefficients and baseline survival estimates. The tabulated probabilities are rounded to three decimal places. For total scores not listed in the table, probabilities should preferably be calculated using the final model equation and the corresponding score-scaling constant rather than by linear interpolation. Linear interpolation may be used only as an approximate clinical presentation aid.

**Supplementary Table S3.** Numbers at risk, recurrence events, and censoring at the prespecified prediction time points

| **Cohort** | **Time point** | **Number at risk** | **Cumulative recurrence events** | **Cumulative censoring** |
| --- | --- | --- | --- | --- |
| Training cohort | 1 year | 160 | 21 | 0 |
|  | 2 years | 139 | 36 | 6 |
| Temporal validation cohort | 1 year | 67 | 8 | 0 |
|  | 2 years | 39 | 15 | 21 |

Note: Number at risk was defined as the number of patients who remained under observation and had not experienced seizure recurrence immediately before the specified time point. Cumulative censoring included patients whose last documented follow-up occurred before the specified time point without a recurrence event. No patients were censored before 1 year because a minimum postoperative follow-up of 1 year was required for study inclusion.
